# Tranexamic acid in the management of traumatic brain injury: a systematic review and meta-analysis with trial sequential analysis

**DOI:** 10.31744/einstein_journal/2025RW0753

**Published:** 2025-02-26

**Authors:** Thiago Gebrin, Júlia Pinho Neodini, André Felix Gentil, Eduardo Carvalhal Ribas, Mario Lenza, Arthur Werner Poetscher

**Affiliations:** 1 Faculdade Israelita de Ciências da Saúde Albert Einstein Hospital Israelita Albert Einstein São Paulo SP Brazil Faculdade Israelita de Ciências da Saúde Albert Einstein, Hospital Israelita Albert Einstein, São Paulo, SP, Brazil.; 2 Hospital Israelita Albert Einstein São Paulo SP Brazil Hospital Israelita Albert Einstein, São Paulo, SP, Brazil.

**Keywords:** Central nervous system, Tranexamic acid, Antifibrinolytic agents, Brain injuries, traumatic, Craniocerebral trauma

## Abstract

**Introduction:**

Traumatic brain injury is a leading cause of death and disability. Tranexamic acid, an antifibrinolytic agent, holds the potential for managing intracranial hemorrhages secondary to traumatic brain injury. However, its efficacy and safety remain subjects of ongoing debate.

**Objective:**

To better clarify the efficacy and safety of tranexamic acid in that context and to evaluate the need for further studies.

**Methods:**

We conducted a comprehensive search of seven electronic databases, eight study repositories, and tertiary sources between January 2021 and 2022 for randomized controlled trials involving victims of traumatic brain injury aged 15 or older who received tranexamic acid versus placebo or standard care. The primary outcomes were all-cause mortality and hemorrhagic complications during treatment. This review incorporated elements of PRISMA guidelines, Cochrane’s Risk of Bias assessment, and GRADE to assess evidence quality. Sensitivity analyses were also conducted.

**Results:**

Out of 6,958 references retrieved, 14 of the 17 randomized controlled trials were analyzed, encompassing a total of 15,017 patients. Analyses for all-cause mortality did not reach statistical significance (RR= 0.95, 95%CI= 0.88-1.02 | trial sequential analysis RR= 0.95, 95%CI= 0.87-1.03). However, the analysis of hemorrhagic complications during treatment showed statistical significance for progressive intracranial hemorrhage (RR= 0.82, 95%CI= 0.68-0.99 | trial sequential analysis RR= 0.82, 95%CI= 0.38-1.78). Analyses of secondary outcomes, namely unfavorable neurological outcome and other adverse effects, did not demonstrate statistical significance.

**Conclusion:**

Tranexamic acid use did not demonstrate efficacy based on all-cause mortality but showed a favorable safety profile. Additional clinical trials may shed light on remaining clinical uncertainties. **Prospero database registration:** CRD42021221949.

## INTRODUCTION

Traumatic brain injury (TBI) and spinal cord trauma are leading causes of death and disability in the context of traumatic lesions.^1^ In 2017, TBI had a global prevalence of 613.49 cases in 100,000 people, representing over 46 million cases, and a global incidence of 283.39 new cases per 100,000 people, accounting for more than 21 million new cases.^2^ Between 2015 and 2017, the United States saw an estimated annual average of 58,512 TBI-related deaths, with 32,547 being unintentional and 25,965 intentional.^3^ The total cost of TBI in the U.S., adjusted for 2019, was estimated at 93 billion dollars.^4^ In 2021, Brazil’s Unified Health System (SUS - *Sistema Único de Saúde*) approved 100,159 hospitalization authorizations for TBI, of which 10,136 (10.12%) resulted in death. The approved production value was nearly 200 million *reais* (almost 40 million dollars as of 2023 currency), with around 100 million *reais* (almost 20 million dollars) allocated for conservative treatment.^5,6^ Given the frequency, severity, and financial impact of TBI, investigating novel therapeutic methods is crucial.

Tranexamic acid (TXA) is a lysine-analog antifibrinolytic agent that inhibits fibrinolysis by reversibly binding to lysine receptor sites on plasminogen and plasmin, thereby disrupting their interaction with fibrin.^7-10^ This drug is considered potentially useful for managing intracranial hemorrhages secondary to TBI. However, its efficacy and safety in this context are not yet well established.^11-14^ While TXA is known for its role in antifibrinolysis, its complete mechanism of action is not fully understood, with evidence suggesting it also reduces the inflammatory response and functions as an immunomodulator.^11,13,15,16^ Nevertheless, TXA’s effect is most pronounced on the day of trauma, indicating that the observed increase in survival rates with timely use is more likely due to its ability to prevent large hemorrhages rather than its potential anti-inflammatory mechanisms.^14^ In the context of TBI, TXA can improve outcomes by preventing large hemorrhages and related complications through the inhibition of fibrinolysis of intracranial blood clots.^17^ However, there is a risk of adverse reactions related to vascular occlusion, such as pulmonary thromboembolism, deep venous thrombosis, and stroke.^17^ Previous meta-analyses have attempted to establish the role of TXA in TBI; however, some^18-23^ did not include recent publications with large sample sizes;^17^ while others lacked a comprehensive literature search, were limited by language restrictions, included quasi-randomized controlled trials (RCTs), and/or did not accept primary studies with control groups receiving standard treatment.^23-26^ Among all the verified meta-analyses,^18-34^ one study^33^ stood out for using trial sequential analysis (TSA);^35^ however, TSA was applied to only one of the evaluated outcomes. Therefore, the available evidence may lack validity. Finally, the high costs of funding clinical trials and the large number of existing meta-analyses on the theme underscore the need to determine whether the required information size (RIS), required sample sizes needed for each outcome’s test statistics to converge towards the true value,^35,36^ have been met with the currently available primary studies.

## OBJECTIVE

To clarify the efficacy and safety profile of tranexamic acid in the management of intracranial hemorrhages secondary to traumatic brain injury and to assess the need for additional clinical trials and meta-analyses. This study will evaluate tranexamic acid’s efficacy based on all-cause mortality. Its safety profile will be assessed considering all-cause mortality, unfavorable neurological outcomes and complications related to the use of the drug. Finally, the need for further studies will be determined by comparing the RIS for each outcome with the sample size available in each analysis.

## METHODS

A systematic review of the literature and meta-analysis with TSA^35^ were performed using CENTRAL, Embase, LILACS, MEDLINE, SciELO, Scopus and Web of Science databases. We also searched the International Clinical Trials Registry Platform, ClinicalTrials.gov, ISRCTN, Brazilian Clinical Trials Registry, Health Canada’s Clinical Trials Database, EU Clinical Trials Register, German Clinical Trials Register and Chinese Clinical Trial Registry. Additionally, we screened lists of references of included articles for analysis and primary studies from other published meta-analyses on the topic. The search strategies used are detailed in the (Table 1S - Supplementary Material - doi.org/10.5281/zenodo.13336291). The review was structured using the patients, intervention, controls, outcomes, study type and publication time interval (PICOST) model.^37^ Originally, the review aimed to include adults (≥18 years), excluding patients with brain death at admission, as outlined in the study protocol available on PROSPERO.^38^ However, upon initial contact with the retrieved studies, it was noted that some relevant papers, selected in previous systematic reviews, included patients under 18 years of age. This led to a modification of the P criteria of PICOST to include patients aged ≥15 years. To minimize bias from this adjustment, a sensitivity analysis was conducted to determine if the changes affected the results. The intervention analyzed was intravenous (IV) TXA compared to placebo or standard treatment described in the studies. The primary outcomes were all-cause mortality and hemorrhagic complications during treatment; secondary outcomes were neurological outcomes at discharge and 6 months later when available, as well as other adverse effects.^18,19,24^ In general terms, outcomes reported by primary studies that could be considered part of or equivalent to the above-mentioned outcomes were deemed valid. Only RCTs, published or not, in any language, available up to the date of the last search (January 2022) were included. The Cochrane Handbook for Systematic Reviews of Interventions was consulted during the study planning and development.^39-41^ This review was guided by the Preferred Reporting Items for Systematic Reviews and Meta-Analyses (PRISMA) 2020.^42-45^ The methodological quality of the included studies was assessed using the Cochrane risk-of-bias evaluation.^46^ Primary studies were deemed to have a high risk of bias if at least one domain was rated as high; an unclear risk of bias if no domain was rated high but at least one domain was rated unclear; and a low risk of bias if all domains were rated as such. The quality of available evidence was evaluated using the Grading of Recommendations Assessment, Development and Evaluation (GRADE) system.^47^ Publication bias was assessed using funnel plots and the linear regression test of funnel plot asymmetry (LRTFPA).

The search for studies, report analysis, risk of bias analysis, and data collection were conducted independently by two junior reviewers. Divergences were resolved through discussion and/or consultation with a third, senior reviewer. Occasional adjustments to the risk of bias assessment and collected data were made collaboratively by two junior reviewers. The GRADE tables were prepared by a junior reviewer and reviewed with a senior reviewer. Authors of primary studies were contacted to obtain reports and address inquiries. Missing data from primary studies, such as standard deviations and event counts, were calculated as needed. All analyses were conducted on a per-protocol basis. Heterogeneity among the included studies was assessed by quantitative means, using heterogeneity tests, including the *I*^2^ index, τ^2^ index, and Cochran’s Q test. Baujat plots are shown to identify studies that contributed disproportionately to heterogeneity in each case. The significance level was set at 5%. Effect estimates for dichotomous variables are presented as risk ratios (RRs), while those for continuous variables were expressed as mean differences, both with 95% confidence intervals (95%CI). All eligible analyses were performed using both fixed- and random-effects models. For eligible syntheses, planned sensitivity analyses were conducted by excluding studies with a high risk of bias and comparing the original and modified P criteria. Additionally, non-planned sensitivity analyses were performed. Non-pre-specified subgroups analyses, whenever pertinent and possible, and meta-regression, in case the databases permit, were also planned to be done. Finally, the RIS for each eligible outcome was determined using TSA.^35^ In case the available sample size for the analysis of an outcome did not meet the RIS, adjusted CI were calculated using TSA.^35^ This method permits to adjust the results in case the RIS is not met, allowing for better control of type I and II errors compared to traditional meta-analysis approaches.^35^ For TSA analyses, it was considered a bi-tailed α=0.05 as type I error probability and β=0.20 as type II error probability.^35^ All possible analyzed outcomes were evaluated using TSA.^35^ A 10% relative risk (RR) reduction was used for all TSA analyses, except for the outcome change in hematoma volume (mL), where empirical mean difference and variance were applied. Zero-event trials were included whenever possible, with a constant of 1.0. The heterogeneity levels among the included primary studies and the proportion of each outcome in the control groups were used, with the former rounded to the nearest integer.^35^ All eligible heterogeneity estimates for the TSA were determined using both inconsistency (*I*^2^) and diversity (*D*^2^) among studies.^35,48^ The publication years of the main reports of the primary studies were considered for these analyses. Results are visually presented as forest plots, funnel plots, Baujat plots, and TSA graphs.

Rayyan,^49,50^ PICO Portal,^51^ Zotero, Mendeley, Microsoft Excel, EndNoteWeb,^52^ Microsoft Forms, The Retraction Watch Database,^53^ Review Manager version 5.4.1, R version 4.1.1^54^ with Meta package,^55^ Copenhagen Trial Unit’s TSA Software,^56^ and GRADEpro GDT^57,58^ were used for study search, reference management, data collection, and statistical analysis. Auxiliary tools included Google Translator,^59^ DeepL Translator,^60^ and Qhanzi.^61^

This review is registered in the PROSPERO database under the code CRD42021221949^38^ (“Use of tranexamic acid in head injury: a systematic revision and meta-analysis with trial sequential analysis”) and in the *Hospital Israelita Albert Einstein* Research Project Management System (*Sistema Gerenciador de Projeto de Pesquisa*) under the code 4505-20.

## RESULTS

**Figure 1:**
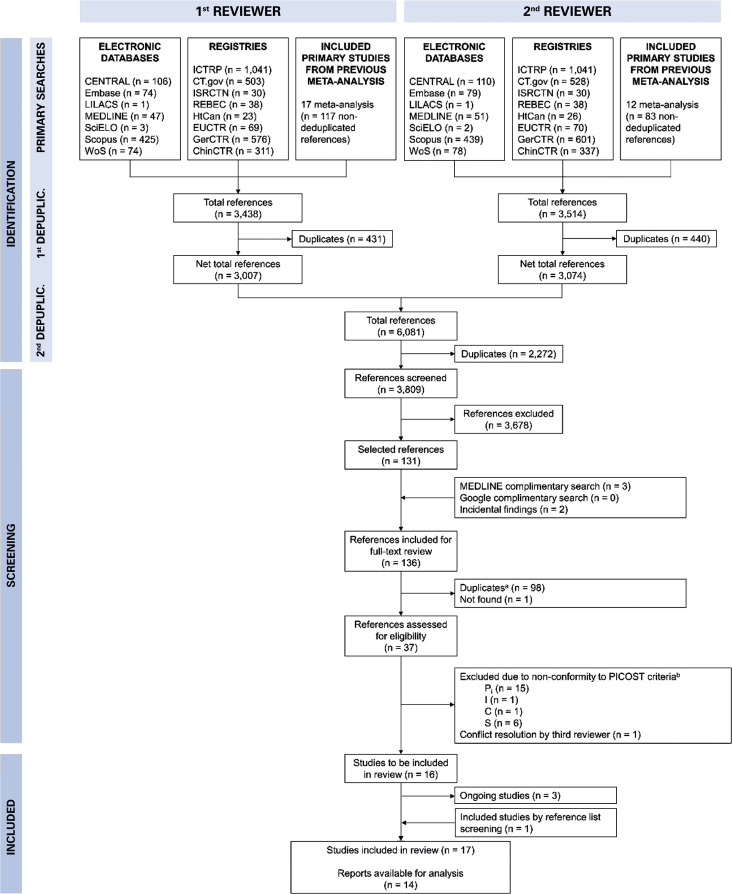
Primary studies selection flowchart ^a^ For each study, publications aside from what has been considered as the main one have also been taken as duplicates.^b^ Total exceeds 20 as some studies were excluded based on more than one PICOST criterion. For the 37 references assessed for eligibility, the preferred PICOST criteria order for study exclusion was SPICO to readily exclude non-randomized clinical trial references. WoS: Web of Science; ICTRP: International Clinical Trials Registry Platform; CT.gov: ClinicalTrials.gov; REBEC: Brazilian Clinical Trials Registry; HtCan: Health Canada’s Clinical Trials Database; EUCTR: EU Clinical Trials Register; GerCTR: German Clinical Trials Register; ChinCTR: Chinese Clinical Trial Registry; P: patients; P_i_: P for inclusion; I: intervention; C: controls; O: outcomes; S: study type; T: publication time interval.

Primary searches were conducted on seven electronic databases, eight repositories and 17 other published meta-analyses^18-34^ between January 2021 and May 2021 by two independent junior reviewers. An update to the primary searches, using only MEDLINE (same search strategy as primary search) and Google (search strategy: “tranexamic acid traumatic brain injury randomized controlled trial”), was conducted between December 2021 and January 2022 by a single junior reviewer. The reference lists of available reports of initially included studies were screened from February 2022 to March 2022 by the same reviewers. A modified PRISMA flowchart is shown in figure 1.

Seventeen studies were included in the review, with 14 having available reports and results at the time of analysis. The included studies are listed in table 1. The three ongoing studies at the time may be valuable for future reviews and meta-analyses. A junior reviewer searched for possible errata, expressions of concern, retractions and others for each included primary study with available reports. This search identified two errata^62,63^ and three commentaries,^64-66^ but no major actions were deemed necessary.

**Table 1:** Characteristics of included studies

Study	RCT characteristics	Demographics		Sample size	ivTXA posology	Outcomes^*^				Declared funding sources	Declared conflicts of interest
		Age^†^	Gender (female)			M	HC	NO	AE		
**Reports with results available at the time**											
CRASH-3 2019^(17)^	Simple randomization Placebo-controlled	43	2,619 (20.6%)	12,737	1 g in 10 min immediately after randomization + 1g in 8 h	✓			✓	National Institute for Health Research Health Technology Assessment, JP Moulton Charitable Trust, Department of Health and Social Care, Department for International Development, Global Challenges Research Fund, Medical Research Council, and Wellcome Trust (Joint Global Health Trials scheme)	None
Atia et al 2021^(95)^	Randomization method is not described Placebo-controlled	29.3	20 (20%)	100	1 g in 10 min + 1 g in 8 h	✓	✓	✓		Not described	Not described
Chakroun-Walha et al 2019^(96)^	Simple randomization Standard treatment-controlled	41±19	17 (9.4%)	180	1 g in 100 mL NS in 10 min + 1g in 500 mL NS in 8 h	✓	✓		✓	None	None
CRASH-2 IBS 2011^(97)^	Nested study Centre-balanced block randomization Placebo-controlled	36.5	42 (15.6%)	270	1 g in 10 min + 1 g in 8 h	✓	✓			CRASH-2: UK NIHR Health Technology Assessment program, Pfizer, BUPA Foundation, J P Moulton Charitable Foundation CRASH-2 IBS: UK Health Technology Assessment program Some authors: UK MRC clinician scientist fellowship, Scottish Funding Council (SINAPSE Collaboration)	None besides the described funding
Ebrahimi et al 2019^(98)^	Simple randomization Placebo-controlled	32.43	12 (15%)	80	1 g in NS 100 mL in 10 min + 1 g in NS 500 mL in 8h	✓	✓			Ahvaz Jundishapur University of Medical Sciences	None
Fakharian et al 2018^(99)^	Block randomization Placebo-controlled	40.8	16 (10.3%)	156	1 g in NS 100 mL in 10 min + 1 g in NS 1000 mL in 8 h	✓	✓	✓		Deputy of Research, Kashan University of Medical Sciences	None besides funding
Fathey et al 2021^(100)^	Simple randomization Placebo-controlled	34.9	12 (30%)	40	1 g in 10 min + 1 g in 8 h		✓		✓	None	None
Jokar et al 2017^(101)^	Simple randomization Placebo-controlled	35.8	20 (25%)	80	1 g in NS 100 mL in 10 min + 1 g in NS 500 mL in 8 h infusion		✓			Arak University of Medical Sciences	Not described
Mojallal et al 2020^(102)^	Simple randomization Placebo-controlled	41±20.27^‡^	20 (20%)^‡^	120	1 g in NS 500 mL in 1 h	✓	✓			Shahid Sadoughi University of Medical Sciences	None
Mousavinejad et al 2020^(103)^	Simple randomization Placebo-controlled	55.02±18.64	14 (35%)	40	1 g in 500 mL NS in 10 min + 1 g in NS 500 mL in 8 h^§^	✓	✓			Ahvaz Jundishapur University of Medical Sciences, Iran	None
Rowell et al 2020^¶(104)^	Block to simple randomization^#^ Placebo-controlled	37.5 (mean of medians)	161 (23.3%)	1,063 (690)	1 g bolus at out-of-hospital setting + 1 g infusion at hospital arrival over 8 h	✓	✓	✓	✓	Series of cooperative agreements from the National Heart, Lung, and Blood Institute administered by the US Army Medical Research & Material Command	Diverse^**^
Safari et al 2021^(105)^	Simple randomization Placebo-controlled	36.3	16 (17%)	94	1 g in first 3 h + 1 g q06h for 48 h		✓	✓		Vice-chancellor of research affairs of the Ahvaz Jundishapur University of Medical Sciences	Not available
Tabesh et al 2016^††(106)^	Block randomization Placebo-controlled	38.1^‡‡^	30 (16%)^‡‡^	190	1 g in NS 100 mL in 10 min + 1 g in NS 100 mL q08h	✓	✓			Research vice-chancellor of Isfahan Medical School	Not described
Yutthakasemsunt et al 2013^(107)^	Block randomization Placebo-controlled	34.5^§§^	28 (11,8%)^§§^	240	1 g in 30 min + 1 g in 8 h^¶¶^	✓	✓	✓	✓	Not clearly described	None
**Ongoing studies at the time**											
EudraCT 2019-004118-33^(108)^	-	-	-	-	-	-	-	-	-	-	-
IRCT2014040915657N1^## (109)^	-	-	-	-	-	-	-	-	-	-	-
IRCT20181225042112N1^## (110)^	-	-	-	-	-	-	-	-	-	-	-

^*^Outcomes refer to those available in each primary study cited publication and collected data. Meta-analysis was not possible in all cases; ^†^ Age in years shown as means ± standard deviation, except where noted; ^‡^ Data refer to a denominator of 100; ^§^In the published article,^(103)^ the maintenance dose of tranexamic acid is described as being administered within 8 min. However, we believe that this was an error and that the correct administration interval probably was 8h. Besides that, the normal saline is cited in some occurrences as 0.09%, which was also considered an error, with the correct concentration being considered as 0.9%. ^¶^ Only data concerning the study groups “tranexamic acid bolus maintenance group,” which received as intervention tranexamic acid only, and “placebo group,” which received as control placebo only, have been considered (690 patients). The study group “tranexamic acid bolus only group” had received both tranexamic acid and placebo and, thus, it had been opted not to include that group in any analyses. Actual study sample size is 1,063; ^#^ In the published article,^(104)^ it is explained that patients were randomly assigned to study groups by a permuted block design of variable block size; however, due mainly to logistic issues, effective randomization was complete instead of a permuted block. ^**^ Description available in the published article;^(104) ††^ The cited report was written in Persian and has been translated by a junior reviewer not fluent in Persian, mainly with translation tools; ^‡‡^ Data refer to a denominator of 95 for the intervention group and 92 for the control group; ^§§^ Data refer to a denominator of 120 for the intervention group and 118 for control group; ^¶¶^ In the published article, the administration method of tranexamic acid is described nowhere. However, it is possible to deduce from the text that it was administered intravenously.

^##^The original hyperlinks for studies IRCT2014040915657N1 and IRCT20181225042112N1 were, respectively, http://www.who.int/trialsearch/Trial2.aspx?TrialID=IRCT2014040915657N1 and http://www.who.int/trialsearch/Trial2.aspx?TrialID=IRCT20181225042112N1, which are currently non-functional and have been replaced by alternative sources.

Numerical data is intention-to-treat.

This table was mainly populated with data from one junior reviewer with punctual inputs from a senior reviewer.

RCT: randomized controlled trial; ivTXA: intravenous tranexamic acid; M, all-cause mortality; HC: hemorrhagic complications during treatment; NO: unfavorable neurologic outcome at discharge and six months later when available; AE: other adverse effects; NS: normal saline; SD: standard deviation.

**Figure 2:**
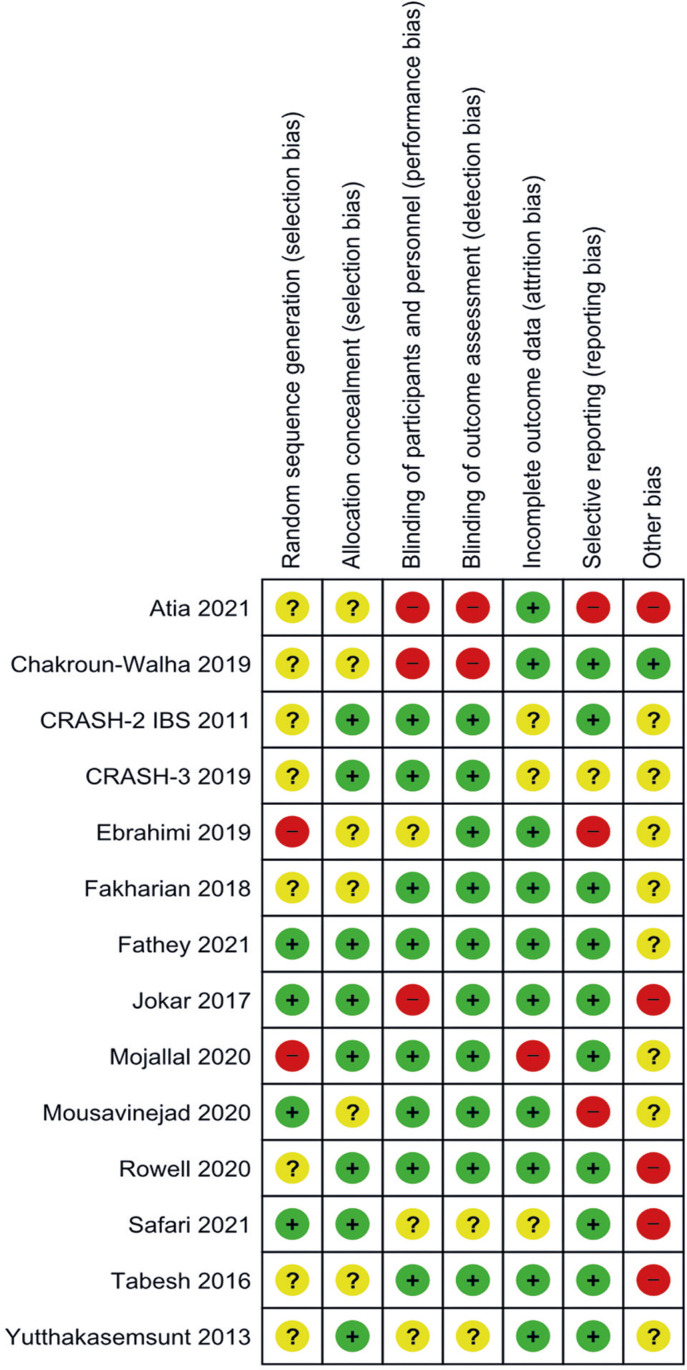
Risk of bias summary

Studies and/or reports were excluded mainly due to duplication (identical reports or secondary reports for the same study) and non-conformity with SPICO criteria (P;^67-78^ I;^79,80^ S^12,16,81-88^. One of the references^89^ with differing opinions from junior reviewers was excluded after consultation with a senior reviewer, as the study authors could not be contacted. Two references^90,91^ were excluded based on criteria P and S; one reference^92^ based on criteria P and C; and reports for two references^93,94^ could not be located with certainty. The Cochrane risk of bias analysis summary and graph for each study are presented in figure 2 and Figure 1S, Supplementary Material (doi.org/10.5281/zenodo.13336291), respectively. Tables 2S-5S, Supplementary Material (doi.org/10.5281/zenodo.13336291), detail the risk of bias analyses for each outcome. It is important to note that not all outcomes and/or primary studies included in the risk-of-bias analyses could be subjected to meta-analysis.

Regarding the criteria “Random sequence generation (selection bias),” most downgrades were due to an inadequate description of the randomization process and baseline differences between groups. The criterion “Allocation concealment (selection bias)” was primarily affected by randomization schemes that could potentially be inferred by study participants, open-labeling, or insufficient reporting in the primary study publication. “Blinding of participants and personnel (performance bias)” was affected by the absence of blinding, insufficient blinding or open-labeling, as well as insufficient reporting. “Blinding of outcome assessment (detection bias)” was influenced by open labeling, insufficient blinding and insufficient reporting. The criteria “Incomplete outcome data (attrition bias)” included issues such as the absence of pre-specified outcomes, incomplete reporting (outcomes not being reported or being reported only by its effect size measures) and reporting of not pre-specified outcomes. Finally, the criteria “Other bias” included conflicts of interest and funding either unstated or stated as present, technical difficulties in applying the intervention and patient cooperation problems (Ebrahimi et al 2019^98^), absence of cited radiologic protocols, with scans apparently reviewed by a single non-radiologist physician (Jokar et al 2017^101^), statistical bias such as type I error from multiple comparisons, exploratory subgroup analyses and potential survival bias (Rowell et al 2020^104^), and limited sample sizes. Classification as unclear or high risk of bias was based on the number of issues in each domain and the author’s judgment.

Table 2 shows the grouping of primary study outcomes in relation to this meta-analysis’ outcomes. Of the 13 outcomes with collected and usable numerical data, three lacked sufficient data for meaningful analyses, resulting in 10 outcomes being analyzed. The statistical analyses for these outcomes are presented below. Subgroup analyses could not be performed due to the limitations of the collected data. Meta-regression was not performed.

**Table 2:** Primary studies’ outcomes grouped as per this meta-analysis outcomes

All-cause mortality	Hemorrhagic complications during treatment	Neurological outcomes at discharge and 6-months later when available*	Other adverse effects
All-cause mortality	Change in hematoma volume (mL) Progressive intracranial hemorrhage Mass effect New hemorrhage Blood product transfusion	Unfavorable neurologic outcome at discharge	Pulmonary embolism Deep venous thrombosis Seizures^†^ Stroke Myocardial infarction^†^ Any thromboembolic event^†^

^*^ There was not sufficient collected data to synthesize neurological outcomes 6 months later; therefore, it is not presented. ^†^ Those outcomes could not be analyzed in a meaningful way and, therefore, are not presented.

### All-cause mortality

**Figure 3:**
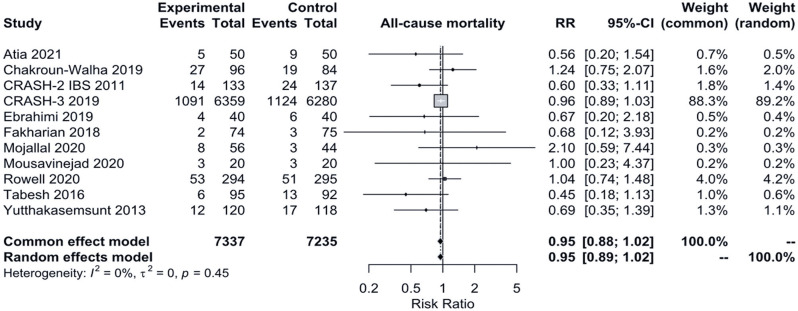
Traditional meta-analysis forest plot for all-cause mortality RR: risk ratio; 95%CI: 95% confidence interval.

The traditional meta-analysis forest plot for all-cause mortality is presented in figure 3. For the CRASH-3 2019 study, events and totals data could not be found in the published article^17^ and were instead derived from presented results utilizing intention-to-treat denominators, as stated in the cited article. All other denominators refer to a per-protocol approach.

Outcome data refer to heterogeneous outcome measurement moments (CRASH-2 IBS 2011, CRASH-3 2019: 28 days post-randomisation, at discharge or at death (whichever first); Mojallal et al 2020: during the first seven days post-admission; Chakroun-Walha et al 2019: at 28th day post-trauma; Atia et al 2021, Ebrahimi et al 2019, Fakharian et al 2018, Mousavinejad et al 2020, Rowell et al 2020, Tabesh et al 2016, Yutthakasemsunt et al 2013: not mentioned).The analysis included 14,572 patients. No significant differences were observed between the fixed- and random-effects models. The fixed-effects model RR= 0.95 (95%CI= 0.88-1.02), indicating no statistically significant difference in all-cause mortality between the groups.

Funnel and Baujat plots are presented in the Figure 2S, Supplementary Material (doi.org/10.5281/zenodo.13336291). LRTFPA was not statistically significant (p=0.3088), indicating no asymmetry and, therefore, no publication bias. The Baujat plot, however, shows that while the CRASH-3 2019 study had a high weight in the overall RR, it had a low influence on overall heterogeneity. Therefore, a sensitivity analysis excluding this study has been performed (Figure 3S, Supplementary Material - doi.org/10.5281/zenodo.13336291), which yielded consistent findings.

**Figure 4:**
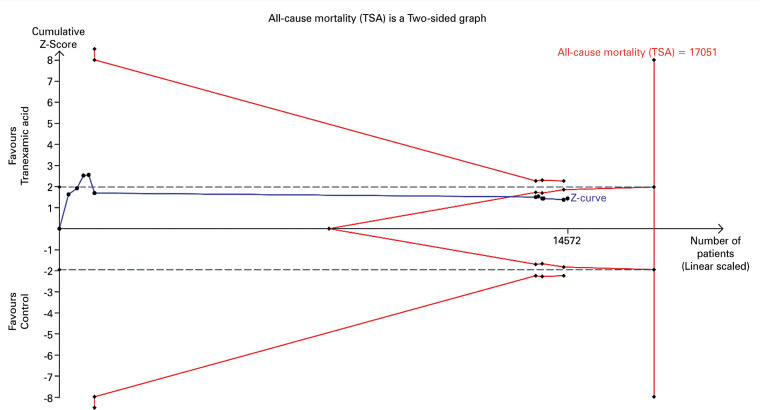
Fixed-effects model trial sequential analysis plot for all-cause mortality Gray lines: Statistical significance boundaries for traditional meta-analysis. Red lines indicate boundaries for statistical significance, futility and required information size. Blue line: Z-curve.

**Figure 5:**
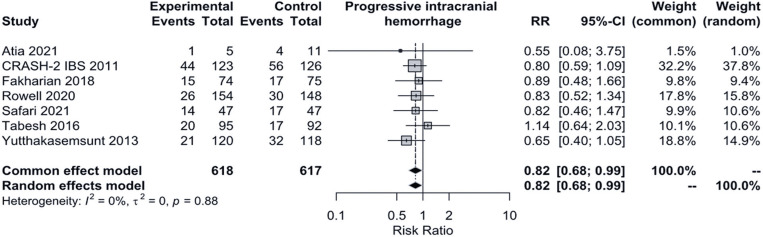
Traditional meta-analysis forest plot for progressive intracranial hemorrhage RR: risk ratio; 95%CI: 95% confidence interval.

A second sensitivity analysis, which included only primary studies with an age inclusion criterion of ≥18 years, was also conducted (Figures 4S and 5S, Supplementary Material - doi.org/10.5281/zenodo.13336291). The results did not change significantly (fixed-effects model RR= 1.06, 95%CI= 0.72-1.56). Due to the insufficient number of primary studies, the LRTFPA could not be performed.

**Figure 6:**
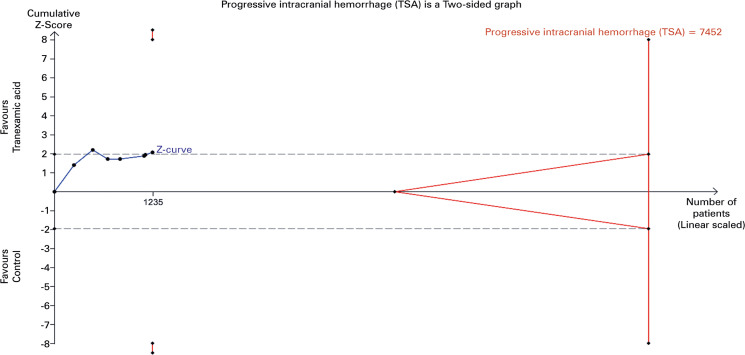
Fixed-effects model trial sequential analysis plot for progressive intracranial hemorrhage Gray lines: Statistical significance boundaries for traditional meta-analysis. Red lines indicate boundaries for statistical significance, futility and required information size. Blue line: Z-curve.

A third sensitivity analysis, which included only primary studies not classified as having a high risk of bias, was also performed (Figures 6S and 7S, Supplementary Material - doi.org/10.5281/zenodo.13336291). The results remained consistent (fixed-effects model RR= 0.95, 95%CI= 0.88-1.02). LRTFPA could not be performed due to the limited number of primary studies.

Since no statistically significant heterogeneity was observed in the traditional meta-analysis (p=0.45), a fixed-effects model TSA analysis is presented (Figure 4). The TSA results similarly showed no statistically significant difference in mortality between groups (RR= 0.95, adjusted 95%CI= 0.87-1.03, Q 9.94 [p=0.4462], *I*^2^ 0%). Despite the available sample size (14,572) being less than the RIS (17,053), the Z-curve crosses the futility boundary, indicating a low probability that additional trials would lead to a statistically significant result (Figure 4).

In summary, our results indicated that TXA did not significantly reduce all-cause mortality in the clinical scenario studied.

### Hemorrhagic complications during treatment

#### Change in hematoma volume (mL)

The traditional meta-analysis forest plot for change in hematoma volume (mL) is shown in Figure 8S, Supplementary Material (doi.org/10.5281/zenodo.13336291). The time intervals for measuring change in hematoma volume varies from 24 to 48 hours, depending on the primary study: 24 hours later for Fathey et al 2021, Mojallal et al 2020, Safari et al 2021, and Tabesh et al 2016; 24 to 48 hours later for Atia et al 2021 and CRASH-2 IBS 2011; and 48 hours later for Jokar et al 2017. It is worth noting that the report for Jokar et al 2017^101^ did not make clear the dispersion measure used for data presentation; thus, we assumed that the standard deviation was employed, as it is among the most commonly used measures and was used in the report elsewhere.

The analysis’s sample size was 850 patients. Neither model showed statistical significance in the mean hemorrhage volume difference between groups (random-effects model MD -1.77mL; 95%CI= -3.83 mL to 0.29 mL).

Funnel and Baujat plots are presented in the Figure 9S, Supplementary Material (doi.org/10.5281/zenodo.13336291). The LRTFPA yielded a p=0.06, indicating a low risk of publication bias. However, the Baujat plot revealed that the Safari et al 2021 study had a strong influence on overall heterogeneity. Therefore, a sensitivity analysis excluding this study was conducted, which resulted in the previously statistically significant heterogeneity (p<0.01) disappearing (p=0.15). The results remained largely unchanged (Figure 10S, Supplementary Material - doi.org/10.5281/zenodo.13336291).

Due to the statistically significant heterogeneity observed in the traditional meta-analysis (p<0.01), Sidik and Jonkman (SJ) and Biggerstaff and Tweedie (BT) random-effects models TSA analyses are presented in the (Figures 11S and 12S, Supplementary Material - doi.org/10.5281/zenodo.13336291). For the SJ random-effects model, the empirical mean difference and variance were -1.6635 and 28.3724, respectively. For the BT random-effects model, the values were -0.6995 and 28.3724, respectively. Standard deviations for the primary studies Fathey et al 2021^100^ and Mojallal et al 2020^102^ were rounded to three decimal places in the TSA analyses. Both models showed no statistically significant differences in the change in hematoma volume between groups (SJ random-effects model: MD -1.66 mL, 95%CI= -5.01mL to 1.68mL, Q 18.1 [p=0.006], *I*^2^ 67%, *D*^2^ 85%; BT random-effects model: MD -0.7mL, 95%CI= -5.56mL to 4.16mL, Q 18.1 [p=0.006], *I*^2^67%, *D*^2^64%).

Therefore, it can be confidently affirmed that the use of tranexamic acid (TXA) did not significantly affect hematoma volume in the studied clinical scenario.

#### Progressive intracranial hemorrhage

The traditional meta-analysis forest plot for progressive intracranial hemorrhage is shown in figure 5. Any documented hemorrhagic growth was considered sufficient to fulfill this outcome. Notably, only some primary studies offered a definition for this outcome in the considered reports. CRASH-2 IBS 2011 classified it as “an increase by ≥25% of total haemorrhage in relation to its initial volume”,^97^ while Rowell et al 2020 defined it as a “>33% increase in the combined volume of subdural, epidural, and intraparenchymal hematomas”,^104^ and Yutthakasemsunt et al 2013 described it as “an intracranial haemorrhage seen on the second CT scan that was not seen on the first CT scan, or an intracranial haemorrhage seen on the first scan that had expanded by 25% or more on any dimension (height, length, or width) on the second scan”.^107^

The sample size for this analysis was 1,235 patients. Both the fixed- and random-effects models produced identical effect point estimates and 95%CI (RR= 0.82, 95%CI= 0.68-0.99). Therefore, the use of TXA significantly reduced the incidence of progressive intracranial hemorrhage, with an 18% reduction in the intervention group compared to the control group (placebo only, in this case).

The Figure 13S, Supplementary Material (doi.org/10.5281/zenodo.13336291), displays the funnel and Baujat plots for the analysis. No statistically significant publication bias was detected in the LRTFPA (p=0.9976).

A sensitivity analysis including only primary studies not classified as having a high risk of bias also showed statistical significance, with an increase in the original protective effect from 18% to 23% (fixed-effects model RR= 0.77, 95%CI = 0.61-0.98), as shown in Figure 14S, Supplementary Material (doi.org/10.5281/zenodo.13336291). LRTFPA could not be performed due to an insufficient number of primary studies (Figure 15S, Supplementary Material - doi.org/10.5281/zenodo.13336291).

As there was no statistically significant heterogeneity in traditional meta-analysis (p=0.88), a fixed-effects model TSA analysis is shown (Figure 6). For this analysis, RR= 0.82, 95%CI= 0.38-1.78, Q 2.42 (p=0.877), I^2^ 0%. In contrast to traditional meta-analysis, TSA results were not statistically significant.

Given these findings, it is prudent to approach the effect of TXA on progressive intracranial hemorrhage with caution. While traditional meta-analyses indicated statistically significant effects, this may be attributable to type I errors (false positives). TSA, which is designed to better control for such errors, did not find any statistically significant effect. Indeed, Figure 6 shows that the Z-curve crosses the conventional statistical significance boundary more than once, reflecting the result of the traditional meta-analysis.

#### Mass effect

The traditional meta-analysis forest plot for mass effect is shown in Figure 16S, Supplementary Material (doi.org/10.5281/zenodo.13336291). It is important to consider that the small number of primary studies may have influenced the validity of these results. In the CRASH-2 IBS 2011 study, mass effect included the evaluation of “(1) sulcal effacement, (2) ventricular effacement, (3) uncal herniation, (4) cisterns compressed, (5) cisterns absent, (6) Midline shift (in mm)”,^97^ while Yutthakasemsunt et al 2013 considered “progressive pressure effect […] as either an increase in midline shift of greater than 1 mm or an increase in basal cistern between the first and second CT scan.”^107^ The Atia et al 2021 study did not specify the outcome.^95^

The sample size for the analysis was 578 patients. Although the random-effects model indicated a statistically significant reduction in mass effect (random-effects model RR= 0.80, 95%CI= 0.64-0.99), as there is no statistically significant heterogeneity (p=0.91), we consider that the fixed-effects model results should be prioritized (fixed-effects model RR= 0.81, 95%CI= 0.65-1.01). Therefore, tranexamic acid seems unable to significantly reduce mass effect frequency between groups.

The Figure 17S, Supplementary Material (doi.org/10.5281/zenodo.13336291), presents the funnel and Baujat plots, with LRTFPA being unperformable due to insufficient primary studies.

Given the lack of statistically significant heterogeneity in the traditional meta-analysis (p=0.91), a fixed-effects model TSA analysis is shown (Figure 18S, Supplementary Material - doi.org/10.5281/zenodo.13336291). The analysis showed no statistical significance (RR= 0.81, 95%CI= 0.33-1.99, Q 0.19 (p=0.9088), *I*^2^ 0%).

Based on these findings, the sample size appears inadequate to draw reliable conclusions. Currently, it seems that tranexamic acid use does not significantly reduce the frequency of mass effect in this clinical scenario.

#### New hemorrhage

The traditional meta-analysis forest plot for new hemorrhage is shown in Figure 19S, Supplementary Material (doi.org/10.5281/zenodo.13336291). The CRASH-2 IBS 2011 study defined new hemorrhage as “new intracranial haemorrhage (apparent on the second scan but not apparent on the first).”^97^ The limited number of primary studies may have impacted the validity of the results.

The sample size for the analysis was 498 patients. Neither the fixed-effects model nor the random-effects model showed a statistically significant difference in new hemorrhage occurrence between groups (fixed-effects model RR= 0.86, 95%CI= 0.56-1.32).

Funnel and Baujat plots are presented in Figure 20S, Supplementary Material (doi.org/10.5281/zenodo.13336291). Due to the small number of primary studies, the LRTFPA test could not be performed.

As the traditional meta-analysis did not indicate statistically significant heterogeneity (p=0.35), a fixed-effects TSA model was prioritized. However, this model could not be executed probably due to the low quantity of information available.

#### Blood products transfusion

The traditional meta-analysis forest plot for blood products transfusion is presented in Figure 21S, Supplementary Material (doi.org/10.5281/zenodo.13336291). The small number of primary studies might have influenced the validity of the results. It is important to address that this outcome initially referred to red blood cell transfusion. The Fathey et al 2021 study provided data for “blood transfusion”, without specifications; thus, it was treated as equivalent to red blood cell transfusion, as whole blood transfusions are uncommon. The Yutthakasemsunt et al 2013 study, in turn, provided data for “blood products” transfusion, which was consequently considered inclusive of any blood products transfusion. Finally, the primary study Rowell et al 2020 provided data both for any blood products transfusion and red blood cell transfusion; however, only data for the former were collected. Therefore, data for Fathey et al 2021 and Rowell et al 2020 refer to red blood cell transfusion, while data for Yutthakasemsunt et al 2013 refer to any blood products transfusion.

The analysis’s sample size was 899 patients. No statistically significant difference in blood products transfusion between the groups was found (fixed-effects model RR= 0.85, 95%CI= 0.66-1.10).

Figure 22S, Supplementary Material (doi.org/ 10.5281/zenodo.13336291), presents the funnel and Baujat plots. An LRTFPA test could not be performed due to the insufficient number of primary studies.

Given the absence of statistically significant heterogeneity in the traditional meta-analysis (p=0.43), a fixed-effects TSA model is shown (Figure 23S, Supplementary Material - doi.org/10.5281/zenodo. 13336291). The TSA analysis similarly revealed no statistically significant difference (RR= 0.85, 95%CI= 0.30-2.41, Q 1.7 (p=0.4267), I^2^ 0%).

## Neurological outcomes at discharge and 6 months later when available

### Unfavorable neurologic outcome (discharge)

The traditional meta-analysis forest plot for unfavorable neurologic outcome (discharge) is shown in the Figure 24S, Supplementary Material (doi.org/10.5281/zenodo.13336291). Primary study Rowell 2020 used the Glasgow Outcome Scale-Extended to measure this outcome, while the other three studies utilized the Glasgow Outcome Scale (Atia et al 2021 did not define the abbreviation “GOS” in the publication,^95^ so it was assumed to refer to the Glasgow Outcome Scale). The small number of primary studies might have affected the validity of the results.

The sample size for this analysis was 1,073 patients. There was no statistically significant difference in neurological outcomes at discharge between the groups, with the fixed-effects model yielding a RR= 0.93 (95%CI= 0.83-1.04).

The Figure 25S, Supplementary Material (doi.org/10.5281/zenodo.13336291), shows funnel and Baujat plots. LRTFPA could not be performed because there were insufficient primary studies.

Due to Rowell et al 2020 using a different scale for measuring this outcome, a sensitivity analysis was conducted excluding that study (Figures 26S and 27S, Supplementary Material - doi.org/10.5281/zenodo.13336291). The analysis showed that the results were not significantly affected (fixed-effects model RR= 0.75, 95%CI= 0.53-1.06). The LRTFPA could not be performed due to the small number of primary studies.

Since there was no statistically significant heterogeneity in the traditional meta-analysis (p=0.54), a fixed-effects TSA model is shown (Figure 28S, Supplementary Material - doi.org/10.5281/zenodo.13336291). The TSA results also showed no statistically significant difference between groups (RR= 0.93, 95%CI= 0.69-1.25, Q 2.43 [p=0.4878], *I*^2^ 0%).

## Other adverse effects

### Pulmonary embolism

A traditional meta-analysis forest plot for pulmonary embolism is shown in Figure 29S, Supplementary Material (doi.org/10.5281/zenodo.13336291). Given the small number of primary studies, the validity of the results may be impacted.

The analysis included a sample size of 13,678 patients. Both the fixed-effects and random-effects models did not show a statistically significant difference in the occurrence of pulmonary embolism between groups (fixed-effects model RR= 0.94, 95%CI= 0.60-1.47).

The Figure 30S, Supplementary Material (doi.org/10.5281/zenodo.13336291), displays the funnel and Baujat plots. Due to the limited number of primary studies, LRTFPA could not be performed.

Since the traditional meta-analysis did not show statistically significant heterogeneity (p=0.06), a fixed-effects TSA model was recommended. However, probably due to insufficient quantity of information, this TSA analysis could not be conducted.

### Deep venous thrombosis

The Figure 31S, Supplementary Material (doi.org/10.5281/zenodo.13336291), includes the traditional meta-analysis forest plot for deep venous thrombosis. It is important to note that the study Fathey et al 2021 reported a combined outcome of pulmonary embolism and deep venous thrombosis. In this particular case, the events and totals presented in the published article^100^ were utilized only for this deep venous thrombosis outcome, as not all patients with deep venous thrombosis progress to pulmonary embolism. Consequently, this study was not included in the former adverse effect analyses. The impact of the small number of primary studies on the results should be considered.

The sample size for this analysis was 13,718. The results showed no statistically significant difference in the incidence of deep venous thrombosis between the groups (fixed-effects model RR= 0.91, 95%CI= 0.54-1.54). Due to the insufficient number of primary studies, LRTFPA could not be performed. Funnel and Baujat plots are shown in Figure 32S, Supplementary Material (doi.org/10.5281/zenodo.13336291). Additionally, probably due to insufficient quantity of information, a fixed-effects TSA model (traditional meta-analysis heterogeneity p=0.33) could not be run.

### Stroke

The traditional meta-analysis forest plot for stroke is shown in the Figure 33S, Supplementary Material (doi.org/10.5281/zenodo.13336291). Given the small number of primary studies, it is important to consider the limitations of the results. The CRASH-3 2019^17^ and Yutthakasemsunt et al 2013^107^ primary studies did not specify the type of stroke, while Rowell et al 2020^104^ considered thrombotic stroke. For the Fathey et al 2021^100^ study, the primary study outcome “Cerebral infarction” was considered equivalent to stroke.

The sample size for the analysis was 13,538 patients. There was no statistically significant difference in stroke occurrence between the groups (fixed-effects model RR= 0.86, 95%CI= 0.60-1.23). Funnel and Baujat plots are available in Figure 34S, Supplementary Material (doi.org/10.5281/zenodo.13336291). The LRTFPA test could not be performed due to the limited number of primary studies.

A sensitivity analysis was conducted focusing only on primary studies not rated as having a high risk of bias (Figures 35S and 36S, Supplementary Material - doi.org/10.5281/zenodo.13336291). The results did not change significantly (fixed-effects model RR= 0.97, 95%CI= 0.66-1.42). LRTFPA could not be performed due to the limited number of primary studies.

Given that there was no statistically significant heterogeneity in the traditional meta-analysis (p=0.14), a fixed-effects TSA model is shown (Figure 37S, Supplementary Material - doi.org/10.5281/zenodo.13336291). The TSA results also showed no statistical difference between groups (RR= 0.86, 95%CI= 0.20-3.76, Q 5.46 [p=0.1411], *I*^2^ 45%).

The Summary of Findings and the GRADE evidence profile tables are presented in Table 3 and Table 6S, Supplementary Material (doi.org/10.5281/zenodo.13336291). Analyses for the composite outcome “Thromboembolic outcomes” (including deep venous thrombosis, pulmonary embolism, and stroke) were conducted separately using a Mantel-Haenszel random-effects traditional meta-analysis, and included data from five studies.^17,96,100,104,107^

**Table 3:** Evidence profile

Certainty assessment						Summary of findings					
Participants (studies) Follow-up	Risk of bias	Inconsistency	Indirectness	Imprecision	Publication bias	Overall certainty of evidence	Study event rates (%)		Relative effect (95% CI)	Anticipated absolute effects	
							With placebo and/or standard treatment	With tranexamic acid		Risk with placebo and/or standard treatment	Risk difference with tranexamic acid
**All-cause mortality**											
14572 (11 RCTs)	serious^a^	not serious^b^	not serious	not serious	none	⨁⨁⨁◯ Moderate	1272/7235 (17.6%)	1225/7337 (16.7%)	RR 0.95 (0.88 to 1.02)	**Study population**	
										176 per 1.000	9 fewer per 1.000 (from 21 fewer to 4 more)
										**Low**	
										50 per 1.000^c^	3 fewer per 1.000 (from 6 fewer to 1 more)
										**High**	
										100 per 1.000^c^	5 fewer per 1.000 (from 12 fewer to 2 more)
**Change in hematoma volume (mL) (follow-up: range 24 hours to 48 hours; assessed with computed tomography imaging)**											
850 (7 RCTs)	serious^d^	serious^e^	not serious	serious^f^	none	⨁◯◯◯ Very low	419	431	-	The mean change in hematoma volume (mL) was 4.17 mL	MD 1.77 mL lower (3.83 lower to 0.29 higher)
**Progressive intracranial hemorrhage (assessed with computed tomography imaging)**											
1235 (7 RCTs)	not serious	not serious^g^	serious^h^	serious^i^	none	⨁⨁◯◯ Low	173/617 (28.0%)	141/618 (22.8%)	RR 0.82 (0.68 to 0.99)	**Study population**	
										280 per 1.000	50 fewer per 1.000 (from 90 fewer to 3 fewer)
										**Low**	
										200 per 1.000^j^	36 fewer per 1.000 (from 64 fewer to 2 fewer)
										**High**	
										360 per 1.000^j^	65 fewer per 1.000 (from 115 fewer to 4 fewer)
**Mass Effect**											
578 (3 RCTs)	serious^k^	not serious^l^	serious^m^	serious^n^	none	⨁◯◯◯ Very low	100/291 (34.4%)	79/287 (27.5%)	RR 0.81 (0.65 to 1.01)	**Study population**	
										344 per 1.000	65 fewer per 1.000 (from 120 fewer to 3 more)
										**Low**	
										100 per 1.000^o^	19 fewer per 1.000 (from 35 fewer to 1 more)
										**High**	
										200 per 1.000^o^	38 fewer per 1.000 (from 70 fewer to 2 more)
**New hemorrhage**											
498 (3 RCTs)	not serious	not serious^p^	not serious	serious^q^	none	⨁⨁⨁◯ Moderate	39/251 (15.5%)	33/247 (13.4%)	RR 0.86 (0.56 to 1.32)	**Study population**	
										155 per 1.000	22 fewer per 1.000 (from 68 fewer to 50 more)
										**Low**	
										100 per 1.000^r^	14 fewer per 1.000 (from 44 fewer to 32 more)
										**High**	
										200 per 1.000^r^	28 fewer per 1.000 (from 88 fewer to 64 more)
**Unfavorable neurologic outcome (discharge) (assessed with Glasgow Outcome Scale and Glasgow Outcome Scale-Extended)**											
1073 (4 RCTs)	serious^s^	not serious^t^	not serious	serious^u^	none	⨁⨁◯◯ Low	253/535 (47.3%)	236/538 (43.9%)	RR 0.93 (0.83 to 1.04)	**Study population**	
										473 per 1.000	33 fewer per 1.000 (from 80 fewer to 19 more)
										**Low**	
										230 per 1.000^v^	16 fewer per 1.000 (from 39 fewer to 9 more)
										**High**	
										340 per 1.000^v^	24 fewer per 1.000 (from 58 fewer to 14 more)
**Thromboembolic complications**											
13718 (5 RCTs)	not serious	serious^w^	serious^x^	not serious^y^	none^z^	⨁⨁◯◯ Low	128/6811 (1.9%)	117/6907 (1.7%)	RR 0.83 (0.44 to 1.56)	**Study population**	
										19 per 1.000	3 fewer per 1.000 (from 11 fewer to 11 more)
										**Low**	
										30 per 1.000	5 fewer per 1.000 (from 17 fewer to 17 more)
										**High**	
										80 per 1.000	14 fewer per 1.000 (from 45 fewer to 45 more)

95% CI: 95% confidence interval; MD: mean difference; RR: risk ratio.

Explanations

a. Out of the 11 randomized controlled trials (RCTs), five (~45%) had at least one domain rated as of high risk of bias, and the remaining six as of unclear risk of bias, in the outcome-level risk of bias assessment. b. Not statistically significant not important heterogeneity (I^2^ = 0%, 𝜏^2^ = 0, p=0.45). c. For this outcome, reasonable thresholds for low (5%) and high (10%) control risks were preferred instead of deriving these risks from the control groups of primary studies. d. Out of the seven RCTs, four (~57%) had at least one domain rated as of high risk of bias, and the remaining three as of unclear risk of bias, in the outcome-level risk of bias assessment. e. Statistically significant substantial heterogeneity (I^2^ = 67%, 𝜏^2^ = 4.5560, p<0.01 | 64% ≤ D^2^ ≤ 85%, p=0.006). f. The required information size was not met (at least 2,231 patients for the Sidik and Jonkman random-effects model), and the available sample size for analysis was low (850 patients). g. Not statistically significant not important heterogeneity (I^2^ = 0%, 𝜏^2^ = 0, p=0.88). h. Not every primary study included in this analysis defined “progressive intracranial hemorrhage,” and the studies that did used different criteria. i. The required information size was not met (at least 7,452 patients), and the available sample size for analysis was moderate (1,235 patients). j. Low (20%) and high (36%) control risks were derived from the proportions of events in the control groups of primary studies. k. Out of the three RCTs, two (~66%) had at least one domain rated as of high risk of bias, and the remaining one as of unclear risk of bias, in the outcome-level risk of bias assessment. l. Not statistically significant not important heterogeneity (I^2^ = 0%, 𝜏^2^ = 0, p=0.91). m. Not every primary study included in this analysis defined “mass effect,” and the studies that did used different criteria. n. The required information size was not met (at least 6,488 patients), and the available sample size for analysis was low (578 patients). o. For this outcome, a reasonable threshold for high (20%) control risk was preferred, while the threshold for low (10%) control risk was derived from the control groups of primary studies. p. Not statistically significant not important heterogeneity (I^2^ = 4%, 𝜏^2^ = 0.0434, p=0.35). q. The required information size was not available; however, the sample size available for this analysis was small (498 patients). r. For this outcome, a reasonable threshold for high (20%) control risk was preferred, while the threshold for low (10%) control risk was derived from the control groups of primary studies. s. Out of the four RCTs, one (25%) had at least one domain rated as of high risk of bias, and the remaining three as of unclear risk of bias, in the outcome-level risk of bias assessment. t. Not statistically significant not important heterogeneity (I^2^ = 0%, 𝜏^2^ = 0.0049, p=0.54). u. The required information size was not met (at least 5,689 patients), and a moderate sample size was available for analysis (1,073 patients). v. Low (23%) and high (34%) control risks were derived from the proportions of events in the control groups of primary studies. w. Statistically significant substantial heterogeneity (I^2^ = 64%, 𝜏^2^ = 0.29, p=0.02). x. As this is a composite outcome. y. The required information size was not available; however, the sample size available for this analysis was large (13,718 patients). z. The presence of publication bias has not been evaluated for this composite outcome.

## DISCUSSION

The use of TXA in trauma management was firmly established following the CRASH-2 trial,^111^ which enrolled over 20,000 adult trauma patients across 274 hospitals in 40 countries. The CRASH-2 trial demonstrated the efficacy of TXA in extracranial trauma, showing a statistically significant reduction in all-cause mortality (9% difference between groups) and mortality due to bleeding (15%). Additionally, it confirmed the drug’s safety, with no significant differences between groups in vascular occlusive events such as any event, myocardial infarction, stroke, pulmonary embolism, or deep venous thrombosis. However, the role of TXA in isolated TBI remained controversial. Numerous clinical trials and systematic reviews have produced conflicting results, and no consensus has been reached. This systematic review and meta-analysis aims to provide more definitive answers to this topic.

The main outcome of this review, all-cause mortality, was not significantly affected by the use of TXA in this clinical scenario under any analyses. This finding is consistent with several reviews^19,23-24,31-32^ but contrasts with those by Chen et al.,^18^ which reported a statistically significant reduction in mortality (RR= 0.91, 95%CI= 0.85-0.97); Du et al.^25^ (RR= 0.92, 95%CI= 0.87-0.96); July et al.^26^ (RR 0.92, 95%CI= 0.88-0.97); and Roberts et al.^21^ (RR= 0.90, 95%CI= 0.85-0.97). To our knowledge, the only other systematic review and meta-analysis incorporating TSA, by Lawati et al.,^33^ also did not demonstrate any significant difference between groups in either the traditional meta-analysis or TSA. Additionally, in our TSA, the Z-curve reached the threshold for futility even before achieving the sample size of 14,572 patients. Therefore, the positive findings reported in the literature may be false positives, as well as the results from some of the primary studies included in the analysis.^99,106^ It is important to highlight, however, that this outcome has not been subjected to subgroup analyses. The CRASH-3 trial^17^ demonstrated the drug efficacy on head-injury-related mortality when administered within three hours of injury to patients with mild-to-moderate TBI (RR= 0.78, 95%CI= 0.64-0.95). Consequently, it is possible that these positive findings from the CRASH-3 trial were diluted in the overall analyses due to the lack of subgroup analyses based on time of administration and injury severity.

Analyses of our second main outcome, hemorrhagic complications during treatment, revealed some statistically significant difference between the groups regarding only progressive intracranial hemorrhage. The traditional meta-analysis indicated an 18% reduction in the incidence of progressive intracranial hemorrhage in the treatment group. This finding was robust, as the risk-of-bias sensitivity analysis demonstrated an even greater protective effect, showing a 23% reduction in the treatment group. However, TSA analyses did not confirm statistical significance (RR= 0.82, 95%CI= 0.38 to 1.78). The traditional meta-analysis result aligns with the findings of Chen et al.^18^ (RR= 0.78, 95%CI= 0.61-0.99), Du et al.^25^ (RR= 0.78, 95%CI= 0.61-0.99), Huang et al.^32^ (RR= 0.78, 95%CI= 0.61-0.99), Gao et al.^29^ (RR= 0.83, 95%CI= 0.74-0.93), Hu et al.^31^ (RR= 0.54, 95%CI= 0.37-0.80), July et al.^26^ (RR= 0.79, 95%CI= 0.64-0.97) and Zehtabchi et al.^23^ (RR= 0.76, 95%CI= 0.58-0.98). To the best of our knowledge, this is the only meta-analysis that has subjected this specific outcome to TSA, which provides better control for type I errors compared to traditional meta-analysis. Given these findings, we opt to consider the effect of TXA on progressive intracranial hemorrhage as uncertain, as the positive findings could be false positives. The other hemorrhagic complications - change in hematoma volume, mass effect, new hemorrhage, and blood products transfusion -, did not show any statistical significance, even after sensitivity and TSA analyses. This lack of significant findings may reflect possible and yet unknown differences in the pharmacodynamics of TXA in central nervous system vessels compared to extracranial vessels. Theoretically, one might expect these outcomes to be more significantly impacted by an antifibrinolytic drug.

The first secondary outcome, unfavorable neurologic outcome at discharge and 6 months later, could only be analyzed at the discharge timepoint. There was no statistically significant difference between groups, aligning with findings from Chen et al.^18^ (RR= 0.72, 95%CI= 0.47-1.11), Du et al.^25^ (RR= 1.01, 95%CI= 0.95-1.06), July et al.^26^ (RR= 0.93, 95%CI= 0.72-1.21), Lawati et al.^33^ (RR= 0.90, 95%CI= 0.69-1.17), Yokobori et al.^24^ (RR= 0.90, 95%CI= 0.61-1.33) and Zehtabchi et al.^23^ (RR= 0.77, 95%CI= 0.59-1.02). Conversely, Huang et al.^32^ reported a statistically significant protective effect with a 25% reduction in unfavorable outcome (RR= 0.75, 95%CI= 0.61-0.93). Data for the 6-month timepoint were available only from Rowell et al 2020,^104^ which did not show a statistically significant difference between groups regarding favorable Glasgow Outcome Scale-Extended (score >4) at this time point (regional site-adjusted difference 2.0, 95%CI= -5.8-9.8).

The final secondary outcome, other adverse effects (which ended up comprising only thromboembolic complications), did not show a statistically significant difference in incidence between the groups. This applies to individual outcomes such as pulmonary embolism, deep venous thrombosis, and stroke, as well as to the combined outcome of thromboembolic complications. This findings align with results from Chen et al.^18^ (pulmonary embolism RR= 1.86, 95%CI= 0.42-8.29, deep venous thrombosis RR= 0.97, 95%CI= 0.64-1.47), Du et al.^25^ (vascular embolism RR= 1.03, 95%CI= 0.71-1.48, stroke RR= 0.60, 95%CI= 0.26-1.42), July et al.^26^ (deep venous thrombosis RR= 0.82, 95%CI= 0.60-1.13, pulmonary embolism RR= 1.00, 95%CI= 0.60-1.66, stroke RR= 0.83, 95%CI= 0.54-1.27, myocardial infarction RR= 0.75, 95%CI= 0.50-1.11, combined outcome RR= 0.85, 95%CI= 0.71-1.02) and Yokobori et al.^24^ (ischemic or thromboembolic complications RR= 1.33, 95%CI= 0.35-5.04). These results underscore the safety profile of TXA regarding the analyzed thromboembolic complications, contrary to concerns based on its mechanism of action.

This study has several strengths and limitations. Key strengths include: the comprehensive and sensitive search strategies, which encompassed major electronic databases, numerous clinical trial registries, and extensive screening of other meta-analyses and included studies, resulting in a total of 6,958 references and 14 primary studies analyzed; the meta-analysis of 10 outcomes; the report of both fixed- and random-effects traditional meta-analysis models; extensive TSA application; and various sensitivity analyses. Among the limitations and challenges, protocol deviations may have introduced bias. The main deviation was the on-the-fly change in the P age criteria of PICOST, for which a sensitivity analysis was performed to control for potential bias introduced by modifying the age criteria. The collected data allowed this analysis only for the all-cause mortality outcome, showing no changes in results between the age strata of 15 years and older versus ≥18 years. Notably, had the inclusion criterion of ≥18 years been maintained, only five of the 14 primary studies could have been analyzed: Atia et al 2021,^95^Chakroun-Walha et al 2019,^96^ Ebrahimi et al 2019,^98^ Mojallal et al 2020,^102^ and Mousavinejad et al 2020.^103^ In summary, the original age criteria would have limited the inclusion to just five primary studies and to analyze only a single outcome, compromising both the comprehensiveness and external validity of the review. Second, TXA’s efficacy was originally meant to be assessed based on head trauma-related mortality and primary studies using only a placebo control; ultimately, all-cause mortality was used, with no differentiation between placebo and standard treatment controls. Additionally, heterogeneity analyses were conducted using only quantitative methods, lacking qualitative approaches. Additionally, meta-regression was not performed to avoid further delaying the study and potentially rendering the searches obsolete (the last update was in January 2022). It was also judged that meta-regression would contribute minimally to the overall findings. Apart from protocol deviations, several other issues should be noted: the translation of a Persian report (Tabesh et al 2016^106^) by a non-fluent reviewer, which may have introduced errors; the complexity of risk-of-bias analyses, admittedly imperfect, which may have impacted the results; procedural and technical difficulties during reference retrieval and management, causing significant delays; potential minor independence issues between reviewers; and technical difficulties with the TSA Software^56^ by the Copenhagen Trial Unit, which occasionally returned apparently inconsistent results, although efforts were made to address these during statistical analyses. In summary, the high complexity and comprehensiveness of this study inevitably contributed to the introduction of errors and biases.

Regarding the need for further studies on this topic, it is noteworthy that the RIS was not achieved in any of the analyses. Only all-cause mortality reached futility boundaries, which *a priori* suggests that additional trials may not be necessary; however, this conclusion should be approached with caution, since possible subgroup positive effects may have not surfaced due to the lacking of subgroup analyses. Besides that, no other analyzed outcome reached futility boundaries. Therefore, we acknowledge the future value of new clinical trials on the topic, particularly those focusing on all-cause mortality and head trauma-related mortality, analyzed under the effects of covariates such as initial neurologic status and time of drug administration. Additionally, further investigation into hemorrhagic complications during treatment, especially change in hematoma volume—which showed a tendency to cross statistical significance boundaries in one of the TSA analysis graphs—and progressive intracranial hemorrhage, which has demonstrated some statistical significance, would be beneficial. The safety profile of TXA has been well-demonstrated both in this review and in other medical literature, thus reducing, in our view, the urgency for trials focusing solely on safety outcomes. Notably, beyond the ongoing studies previously reported, a new trial protocol^112^ has been published, which, once completed, may provide valuable data for inclusion in future reviews.

## CONCLUSION

Tranexamic acid use did not show efficacy based on all-cause mortality in the management of intracranial hemorrhages secondary to traumatic brain injury. However, it demonstrated an adequate safety profile with respect to all-cause mortality, unfavorable neurologic outcome at discharge and serious thromboembolic complications. Additional clinical trials may shed light on remaining clinical uncertainties.
